# Biomechanics of Human Plantar Skin: Experimental and Constitutive Analysis

**DOI:** 10.1007/s10439-026-04050-8

**Published:** 2026-03-14

**Authors:** Chiara Giulia Fontanella, Sofia Pettenuzzo, Alice Berardo, Elisa Belluzzi, Assunta Pozzuoli, Pietro Ruggieri, Emanuele Luigi Carniel

**Affiliations:** 1 Department of Industrial Engineering, University of Padova, Padua, Italy; 2 Centre for Mechanics of Biological Materials, University of Padova, Padua, Italy; 3 Department of Civil, Environmental and Architectural Engineering, University of Padova, Padua, Italy; 4 Musculoskeletal Pathology and Oncology Laboratory, Department of Surgery, Oncology and Gastroenterology (DiSCOG), University of Padova, Padova, Italy; 5 Orthopedics and Orthopedic Oncology, Department of Surgery, Oncology and Gastroenterology (DiSCOG), University-Hospital of Padova, Padua, Italy

**Keywords:** Foot, Human plantar skin, Mechanical tests, Constitutive model, Ex vivo analysis

## Abstract

**Purpose:**

Plantar skin is a highly specialised tissue which protects the foot from injuries and adapts to external stresses. However, it can be subjected to diabetic plantar ulcers, which are among the most difficult and costly wounds to treat. Although this is a crucial topic, few studies have focused on the mechanical properties of foot skin and how disease alters them. In this context, this work aims to fully describe the mechanical behavior of plantar skin through experiments and constitutive analysis.

**Methods:**

Different experimental tests (failure tensile tests, unconfined compression at different strain rates, stress relaxation tests) were conducted on human plantar skin samples cut along the posterior-anterior (PA), lateral-medial (LM), and cranial-caudal (CC) directions. Then, experimental results were used to identify, through an inverse analysis, the parameters of the anisotropic visco-hyperelastic constitutive model adopted to describe the skin's mechanical response.

**Results:**

Plantar skin’s non-linear, anisotropic, and time-dependent behavior, with differences between the anterior and posterior foot’s regions. In addition, the constitutive model adopted is able to capture the mechanical behavior of the plantar skin Failure tensile tests showed that PA directions exhibited higher elastic modulus than LM directions in both posterior (22.05 vs 12.91 MPa) and anterior (17.39 vs 12.82 MPa) regions, while the unconfined compression tests revealed that compressive elastic moduli in the posterior region increased with increasing strain rates.

**Conclusion:**

The proposed model provides new insights into the mechanics of plantar skin, being a valuable tool for applications such as diagnosing skin diseases and developing skin substitutes.

## Introduction

The skin covers the human body and is a complex living material with several key functions, including regulating body temperature, protection, excretion, and absorption. In terms of its structural configuration, it is primarily composed of two layers, the epidermis and the dermis. The epidermis is mainly composed of epithelial cells, while the dermis contains cells and fibrous proteins, such as collagen and elastin. These proteins form a dense network of fibers and fiber bundles embedded within a ground substance. The skin covering the soles of the human feet, or plantar skin, is highly specialized, and its main functions are to protect the body from injuries and adapt to external stresses during daily activities [1, 2]. It is thicker than other body areas, particularly regarding the stratum corneum, and presents an accelerated epidermal turnover rate with a complete lack of hair and sebaceous glands. The collagen fibers are thicker than in non-plantar skin and preferentially oriented tangentially to the epidermis [3].

This network of thick fibers running parallel to the epidermis may protect plantar skin from excessive loads, enhancing its resistance and stiffness [3]. Nevertheless, skin injuries, such as pressure ulcers or age-associated skin tears, are frequent and common. The disease that most impacts on plantar skin, and in general on foot soft tissues, is the diabetes mellitus. Diabetes mellitus is a systemic disease, with a high social and economic impact affecting more than 400 million people worldwide. Diabetes foot problem is the formation of Diabetic Foot Ulcer (DFU) that contributes to significant morbidity and mortality due to repeated hospitalization and the risk of amputation [4]. Moreover, DFUs are one of the most challenging and costly wound types to manage [4–6]. Understanding the mechanical properties of human skin is crucial for various applications, including the diagnosis and monitoring of skin diseases, the design and development of skin substitutes, and the quantification of the load tolerance of skin, which is a key factor in preventing skin injuries [7]. Several experimental techniques, such as indentation, suction, and uniaxial tensile tests, are commonly used to characterize the mechanical response of the skin, highlighting its anisotropic, non-linear and time-dependent nature [8]. Despite extensive research on human skin, there are very few studies that focus on the mechanical properties of plantar skin. Among the available studies, some examine the effects of diabetes, revealing varying mechanical responses in terms of elastic (compressive and shear) and viscoelastic properties. However, these findings are largely limited to the posterior region of the foot [9, 10] or are based on cadaveric specimens [10].

When considering the skin in the context of a fiber-reinforced composite material, the ground substance serves as the isotropic matrix and the fibrous proteins, particularly collagen, act as the reinforcing fibers. Langer was the first to notice the natural orientation of collagen fibers [11, 12]. He observed that when circular holes were punched in cadaver skin, they enlarged and became elliptical due to the predominant pre-stress along and across the major axes of the ellipse. While Langer's lines are the most well-known skin tension lines, other lines have also been proposed [13]. In general, all distributions reported in the literature indicate that the orientation of collagen fibers depends on the specific location within the body, influenced by the motion of nearby joints or structures. Regarding the skin of the foot, various orientations of collagen fibers have been assessed across different regions of the foot, with a not clear identification of collagen orientation [14, 15]. In addition, considering previous works, the epidermis provides the whole skin the elastic rigidity while the dermis ensures the viscoelastic behavior [16].

Recently, various approaches have been explored to develop a skin substitute for treating skin pathologies, particularly those related to DFU [17, 18]. The existing approaches provided different techniques, from the synthetic materials (e.g., polyvinyl alcohol (PVA) gels or silicone) to the scaffold for cell growth and proliferation [19]. Thus, the complete and specific characterisation of the mechanical behavior of the plantar skin is crucial for developing a skin substitute mimicking plantar skin properties and to develop in silico models based on the Finite Element Method (FEM) as a robust tool for investigating the biomechanics of foot skin, also concerning DFU prevention or treatment [20, 21]. The development of a realistic FE foot model capable of accurately representing the behavior of foot skin is essential, as these models are a valuable tool for obtaining information on, e.g., stress/strain distribution, pressure fields, contact forces within different tissues that cannot be obtained through in vivo or ex vivo tests due to ethical, technical or practical constraints. Several FE models of the human foot have been developed, reflecting different assumptions about constitutive formulations and constitutive parameters adopted [20]. Most of all applied an isotropic elastic or hyperelastic formulation to characterize the mechanical behavior of the skin [22, 23] or including anisotropy while omitting the viscoelastic contribution [24].

In this context, this work aims to fully describe the mechanical behavior of human plantar skin through experimental tests and constitutive analysis. In order to comprehensively characterize skin mechanics, a broad campaign of experimental tests was developed. Unconfined compression tests were performed along the skin thickness direction (${\mathbf{a}}_{0}$, also referred to as cranial-caudal - CC), while tensile tests were carried out along the posterior-anterior (${\mathbf{b}}_{0}$, PA) and lateral-medial (${\mathbf{c}}_{0}$, LM) directions. These three directions considered, ${\mathbf{a}}_{0}$, ${\mathbf{b}}_{0}$, ${\mathbf{c}}_{0}$, form an orthogonal reference system.

## Materials and Methods

### Experimental Investigation of Skin Mechanics

**Fig. 1:**
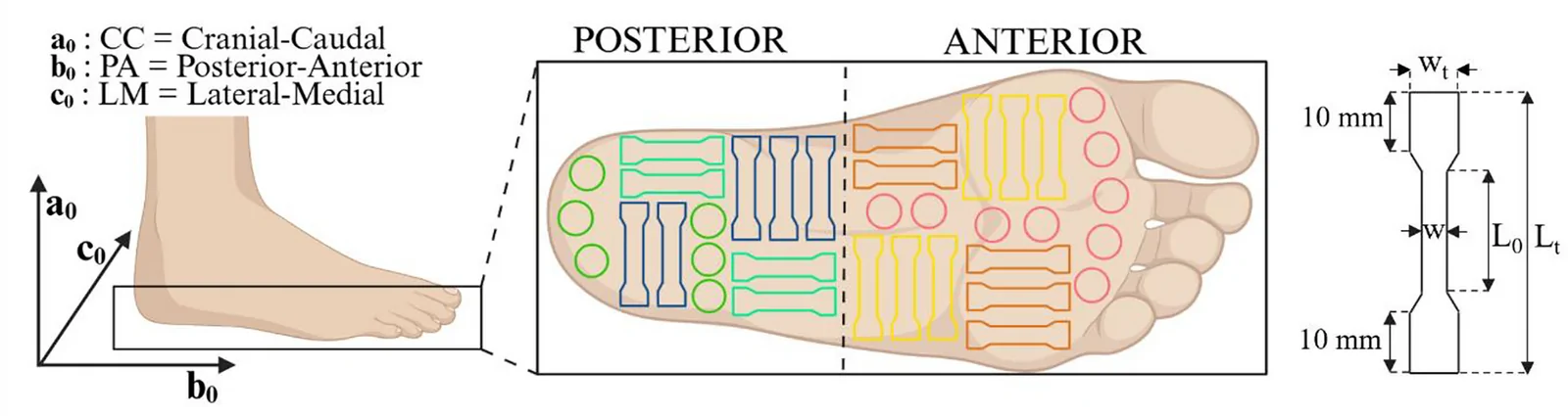
Harvesting of tissue samples and identification of the cranial-caudal (CC,${\mathbf{a}}_{0}$), posterior-anterior (PA, ${\mathbf{b}}_{0}$) and lateral-medial (LM, ${\mathbf{c}}_{0}$ ) directions. On the right, geometry of the dog-bone shaped samples, where w = width = 4.35 ± 0.91 mm; w_t_ = total width = w + 4 mm; L_0_ = length = 19.86 ± 3.95 mm; L_t_ = total length = L_0_ + 25 mm

Plantar skin was collected from male human donors, (4 males; 56 ± 17 years; Body Mass Index 26.1 ± 2.1 kg/m^2^) who underwent amputation due to leg cancer at the Orthopedics and Orthopaedic Oncology Unit, University Hospital of Padova (CESC Code: AOP2649). More precisely, the tumour was located and confined far from the foot (pelvis, thigh, or distal femur), with no anatomical, vascular, or surgical continuity with the distal leg or plantar region, thereby minimising any potential pathological influence. In addition, none of the patients received systemic pharmacological treatments (chemotherapy, immunotherapy, or radiotherapy). For these reasons, the plantar skin tested was considered healthy. All patients gave their written informed consent to be included in the study. After amputation, the plantar foot was delivered to the Laboratory of Mechanics of Biological Materials of the University of Padova and stored at − 20 °C until dissection [25]. After defrosting, the skin was dissected, divided into anterior and posterior regions, and washed with cold saline to eliminate tissue debris and blood residuals. After this procedure, the plantar skin was manually trimmed into dog-bone shaped samples cut according to PA (**b**_0_) and LM (**c**_0_) directions (length = 19.86 ± 3.95 mm; thickness = 2 ± 0.39 mm; width = 4.35 ± 0.91 mm), or cut into cylindrical samples along CC (**a**_0_) direction using a 5 mm punch (height 2.33 ± 0.65 mm). Sizes (length, thickness, width and height) were measured for each sample using a manual calliper. 189 samples were obtained from the different foot regions: 84 from the anterior and 105 from the posterior regions (an example is reported in Fig. 1). Mechanical tests were performed at room temperature within a few hours of thawing. All samples were kept moist in saline solution before immediate testing to preserve hydration [9]. In addition, during experimental tests, sample hydration was maintained by applying saline solution with a pipette at intervals of no more than 300 s to prevent the samples' surfaces from drying.

Mechanical tests were performed with the Biomomentum testing machine (Model Mach-1 v500csst, ©Biomomentum Inc.) using the uniaxial load cell with a capacity of 250 N and resolution of 0.0125 N. The dog-bone shaped samples were clamped by C-shaped tensile grips with textured pattern surfaces. To avoid axial slippage, ensuring effective load transmission along the loading direction, each end of the samples was interposed and glued (Glue Loctite 4013 Prism, Hisco, Houston, TX, USA) between two small Velcro strips [26, 27]. For uniaxial compression tests, the testing machine was equipped with a stainless steel 316L flat indenter, directly screwed into the load cell, with a diameter of 31.75 mm, and with a stainless steel 316L plate above where the sample was positioned for the test. This condition ensures minimal friction at the indenter–specimen interface, allowing lateral expansion and approximating a predominantly uniaxial stress state. Before the mechanical tests, a contact criterion of 0.1 N was applied to each specimen to prevent slack or misalignment of the sample (for tensile tests) and to ensure contact between the flat indenter and the specimen (for compression tests). Following this step, all samples underwent a preconditioning phase consisting of 10 cycles of sinusoidal displacement, with a total applied strain of 5% and a frequency of 1 Hz. The number of preconditioning cycles was selected based on previous studies of collagen-rich soft connective tissues comparable to skin [28, 29], as well as on the analysis of the force–time response during preconditioning, which was shown to be stable after the seventh cycle [30]. This phase is crucial because it stabilises tissue behaviour and reduces the influence of the plantar skin’s unknown prior loading history by inducing progressive microstructural changes through the preconditioning strain. Then, the tissue was allowed to recover during a resting phase of 30 s before starting the effective test.

Uniaxial tensile tests up to failure or stress–relaxation (SR) tests were performed on samples cut according to the PA (${\mathbf{b}}_{0}$) and LM (${\mathbf{c}}_{0}$) directions, while uniaxial unconfined compression tests at different strain rates or SR tests were performed on cylindrical samples obtained along the CC (${\mathbf{a}}_{0}$) direction following the protocol reported in Table 1.

**Table 1:** Protocol parameters for each experimental test

	tensile tests up to failure	tensile SR	unconfined compression LU	unconfined compression SR
Posterior region, No	20 (PA) 30 (LM)	9 (PA) 8 (LM)	14 (${\dot{\varepsilon }}_{1})$ 10 (${\dot{\varepsilon }}_{2}$)	14
Anterior region, No	17 (PA) 20 (LM)	8 (PA) 9 (LM)	11 (${\dot{\varepsilon }}_{1})$ 7 (${\dot{\varepsilon }}_{2}$)	12
Testing protocol parameters	$\dot{\varepsilon }$= 1%s^−1^	5 ramps ${\varepsilon }_{SR} =7$% ${\dot{\varepsilon }}_{SR}=10$%s^−1^ τ = 600 s	${\varepsilon }_{LU} = 5$ 0% ${\dot{\varepsilon }}_{1}=$ 1%s^−1^ ${\dot{\varepsilon }}_{2}=$ 100%s^−1^	5 ramps ${\varepsilon }_{SR} =10$% ${\dot{\varepsilon }}_{SR}=10$%s^−1^ τ = 600 s

No, number of samples; SR, stress-relaxation; LU, loading-unloading; PA, posterior-anterior; LM, lateral-medial.

The choice of testing procedure, based on the sample direction, was determined by the skin structure. It consists of a thick layer of dermis overlain by a thin layer of epidermis. In terms of the properties of the two tissues and their relative thickness, the mechanical behavior is predominantly determined by the dermis, which is a dense connective tissue consisting of an isotropic ground substance within which collagen and elastin fibres are distributed. Concerning collagen fibres, it is essential to notice that they are mainly contained in the skin plane [7] and in plantar skin dermis, where they are oriented tangentially to the epidermis [3]. Therefore, tensile tests were performed on samples lying in the skin plane but cut in two different directions (PD and LM) to assess the contribution of the collagen fibres. In contrast, compression tests were conducted on samples taken perpendicular to the skin plane to evaluate the ground substance matrix properties.

**Fig. 2:**
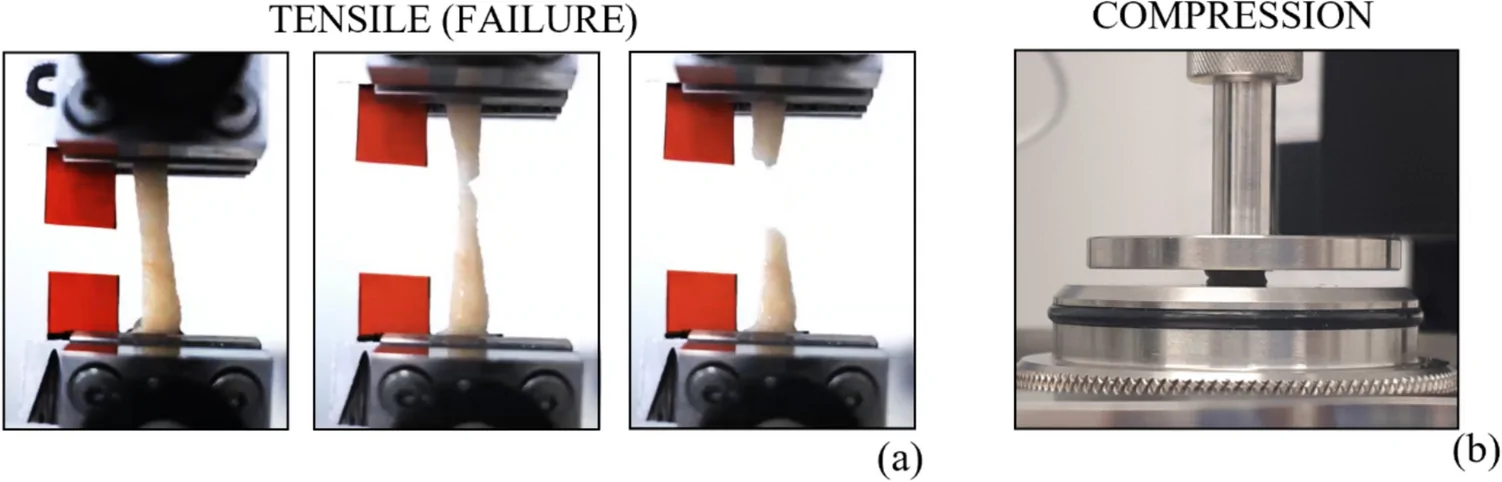
Experimental setup: **a** Tensile test up to failure, showing the different sample conditions at the beginning of the test, during failure, and after rupture; **b** Unconfined compression test

In detail, samples were tested up to the failure by applying a progressive strain with a strain rate of 1%s^−1^. The testing procedure was stopped when a decreasing force was measured in correspondence with visible macroscopic sample breaking (Fig. 2a). Sample failure usually occurred within the central section of the dog-bone–shaped specimens, in agreement with the expected stress distribution in dog-bone geometries, where the smaller cross-section encourages a more uniform axial stress distribution and localises the maximum tensile stress away from the clamping region. The stress–relaxation protocols included 5 ramps of specific strain at a 10%s^−1^ strain rate, followed by 600 s of resting, to allow the almost complete development of relaxation phenomena. Unconfined compression (Fig. 2b) tests up to the 50% strain at two different strain rates (1%s^−1^ or 100%s^−1^) were carried out. Eight loading-unloading (LU) cycles were performed for each strain rate, and the last loading curve corresponding to a stable response was considered. Considering the unconfined compression stress-relaxation protocol, 5 ramps of 10% strain each were realized at a 10% s^-1^ strain rate, followed by 600 s of resting time.

### Constitutive Analysis of Skin Mechanics

The constitutive analysis of the biological tissue began with the mathematical definition of the constitutive model, based on the tissue microstructure and data from experimental activities. The following step was to identify the constitutive parameters by the inverse analysis of experimental tests, such as equilibrium stress-strain and relaxation curves. Finally, the reliability of the model and associated constitutive parameters was assessed by direct analysis of further experimental activities, such as constant strain rate tests [31, 32].

#### Formulation of the Anisotropic and Visco-Hyperelastic Constitutive Model

The mechanical behavior of the biological tissue under investigation, as previously described in Section "Experimental Investigation of Skin Mechanics", is predominantly determined by the dermis. Indeed, the isotropic properties are determined by the isotropic ground substance evaluated considering compression tests, while the anisotropic ones are conferred by the collagen fibres contained in the skin plane [7] and oriented tangentially to the epidermis [3], evaluated by tensile tests. Concerning the principal directions of anisotropy, their identification was based on an engineering approach, using testing along two directions (plus the thickness direction) and developing an anisotropic model based on these directions, an approach previously adopted for other tissues [33]. The definition of the constitutive model, therefore, required an investigation of the deformation mechanisms by which the tissue responds to mechanical stimuli. These mechanisms coincide mainly with the processes of uncrimping of collagen fibrils and recruitment of inter- and intra-fibrillar bonds, stretching of protein chains within elastin fibers and recruitment of chemical bonds, as far as the tensile response is concerned, which is characterized by the typical J-shaped trend of the stress-strain curve. The compressive response is mainly due to the packing of the different constituents and the redistribution of the ground substance within the fibrous solid skeleton. These mechanisms are time-dependent and result in different degrees of stiffness depending on the loading time.

The mechanical behavior of the dermis is therefore characterized by large deformations, anisotropy, non-linear elasticity and time dependence. It was consequently necessary to formulate the model in the framework of anisotropic visco-hyperelasticity [34]:

1 $${\mathbf{S}}\left( {{\mathbf{C}},{\mathbf{q}}^{i} } \right) = 2\frac{{\partial W\left( {\mathbf{C}} \right)}}{{\partial {\mathbf{C}}}} - \sum\limits_{i = 1}^{n} {{\mathbf{q}}^{i} } = {\mathbf{S}}^{0} \left( {\mathbf{C}} \right) - \sum\limits_{i = 1}^{n} {{\mathbf{q}}^{i} }$$

where ${\mathbf{S}}$ is the second Piola-Kirchhoff stress tensor, ${\mathbf{C}}$ is the right Cauchy-Green strain tensor, ${\mathbf{q}}^{i}$ are viscous variables. W is a hyperelastic potential that represents the instantaneous mechanical response of the tissue, which refers to the behavior of the material when there is no time for time-dependent deformation mechanisms to develop, i.e., when the mechanical stimulus is applied at a sufficiently high rate. ${\mathbf{S}}^{0}$ is the instantaneous stress corresponding to the strain ${\mathbf{C}}$. The evolution of the viscous variables is governed by the following differential equations [35]:

2 $${\dot{\mathbf{q}}}^{i} + \frac{1}{{\tau^{i} }}{\mathbf{q}}^{i} = \frac{{\gamma^{i} }}{{\tau^{i} }}{\mathbf{S}}^{0}$$

where $\tau^{i}$ is the relaxation time and $\gamma^{i}$ is the relative stiffness, characterizing the timing and the stiffening contribution of the *i*th deformation mechanism. Relative stiffnesses allow the instantaneous mechanical response to be related to the equilibrium response:

3 $$W^{\infty } \left( {\mathbf{C}} \right) = \left( {1 - \sum\limits_{i = 1}^{n} {\gamma^{i} } } \right)W\left( {\mathbf{C}} \right)$$

4 $${\mathbf{S}}^{\infty } \left( {\mathbf{C}} \right) = 2\frac{{\partial W^{\infty } \left( {\mathbf{C}} \right)}}{{\partial {\mathbf{C}}}} = 2\left( {1 - \sum\limits_{i = 1}^{n} {\gamma^{i} } } \right)\frac{{\partial W\left( {\mathbf{C}} \right)}}{{\partial {\mathbf{C}}}} = \left( {1 - \sum\limits_{i = 1}^{n} {\gamma^{i} } } \right){\mathbf{S}}^{0} \left( {\mathbf{C}} \right)$$

where $\gamma^{\infty } = 1 - \sum\limits_{i = 1}^{n} {\gamma^{i} }$ is the equilibrium relative stiffness.

The formulation of the model therefore required the hyperelastic potential W to be defined [36]:

5 $$W\left( {\mathbf{C}} \right) = U_{iso} \left( J \right) + \tilde{W}_{iso} \left( {\tilde{I}_{1} } \right) + W_{b} \left( {I_{4} } \right) + W_{c} \left( {I_{6} } \right)$$

where $J = \sqrt {\det \left( {\mathbf{C}} \right)}$ is the deformation Jacobian, $\tilde{I}_{1} = J^{ - 2/3} tr\left( {\mathbf{C}} \right)$ is the first isochoric invariant of the right Cauchy-Green strain tensor, $I_{4} = {\mathbf{b}}_{0} \cdot {\mathbf{Cb}}_{0}$ and $I_{6} = {\mathbf{c}}_{0} \cdot {\mathbf{Cc}}_{0}$ are structural invariants equal to the square of the tissue stretch along directions ${\mathbf{b}}_{0}$ and ${\mathbf{c}}_{0}$ [35]. $U_{iso}$ specifies the isotropic and volumetric contribution to the hyperelastic potential, $\tilde{W}_{iso}$ the isotropic and isochoric contribution, while $W_{b}$ and $W_{c}$ define the anisotropic terms:

6 $$U_{iso} \left( J \right) = \frac{K}{{2 + r\left( {r + 1} \right)}}\left[ {\left( {J - 1} \right)^{2} + J^{ - r} + rJ - \left( {r + 1} \right)} \right]$$

where K is the bulk modulus of the tissue in the undeformed configuration [37], and r indicates the volumetric stiffening with strain;

7 $$\tilde{W}_{iso} \left( {\tilde{I}_{1} } \right) = \frac{G}{{2\alpha_{1} }}\left\{ {\exp \left[ {\alpha_{1} \left( {\tilde{I}_{1} - 3} \right)} \right] - 1} \right\}$$

where G is the shear modulus of the tissue in the undeformed configuration, and $\alpha_{1}$ specifies the isotropic stiffening with strain;

8 $$W_{b} \left( {I_{4} } \right) = \frac{{C_{4} }}{{\alpha_{4}^{2} }}\left\{ {\exp \left[ {\alpha_{4} \left( {I_{4} - 1} \right)} \right] - \alpha_{4} \left( {I_{4} - 1} \right) - 1} \right\}$$

9 $$W_{c} \left( {I_{6} } \right) = \frac{{C_{6} }}{{\alpha_{6}^{2} }}\left\{ {\exp \left[ {\alpha_{6} \left( {I_{6} - 1} \right)} \right] - \alpha_{6} \left( {I_{6} - 1} \right) - 1} \right\}$$

where $C_{4}$ and $C_{6}$ are related to the initial longitudinal stiffness of the tissue along directions ${\mathbf{b}}_{0}$ and ${\mathbf{c}}_{0}$, respectively, while $\alpha_{4}$ and $\alpha_{6}$ are anisotropic stiffening parameters.

Given the complexity of the biological tissue conformation and mechanics, different viscous variables were associated with the isotropic term, such as ${\mathbf{q}}_{iso}^{i}$, and the two anisotropic contributions, such as ${\mathbf{q}}_{b}^{i}$ and ${\mathbf{q}}_{c}^{i}$:

10 $$\begin{aligned} {\mathbf{S}}\left( {{\mathbf{C}},{\mathbf{q}}^{i} } \right) & = {\mathbf{S}}_{iso} \left( {J,\tilde{I}_{1} ,{\mathbf{q}}_{iso}^{i} } \right) + {\mathbf{S}}_{b} \left( {I_{4} ,{\mathbf{q}}_{b}^{i} } \right) + {\mathbf{S}}_{c} \left( {I_{6} ,{\mathbf{q}}_{c}^{i} } \right) \\ & \; = \left\{ {{\mathbf{S}}_{iso}^{0} \left( {J,\tilde{I}_{1} } \right) - \sum\limits_{i = 1}^{{n_{iso} }} {{\mathbf{q}}_{iso}^{i} } } \right\} + \left\{ {{\mathbf{S}}_{b}^{0} \left( {I_{4} } \right) - \sum\limits_{i = 1}^{{n_{b} }} {{\mathbf{q}}_{b}^{i} } } \right\} + \left\{ {{\mathbf{S}}_{c}^{0} \left( {I_{6} } \right) - \sum\limits_{i = 1}^{{n_{c} }} {{\mathbf{q}}_{c}^{i} } } \right\} \\ \end{aligned}$$

11 $$\begin{aligned} {\mathbf{S}}_{iso}^{0} \left( {J,\tilde{I}_{1} } \right) & = 2\frac{{\partial U_{iso} }}{{\partial {\mathbf{C}}}} + 2\frac{{\partial \tilde{W}_{iso} }}{{\partial {\mathbf{C}}}} \\ & \; = 2\frac{{\partial U_{iso} }}{\partial J}\frac{\partial J}{{\partial {\mathbf{C}}}} + 2\frac{{\partial \tilde{W}_{iso} }}{{\partial \tilde{I}_{1} }}\frac{{\partial \tilde{I}_{1} }}{{\partial {\mathbf{C}}}} = \frac{{\partial U_{iso} }}{\partial J}J{\mathbf{C}}^{ - 1} + 2\frac{{\partial \tilde{W}_{iso} }}{{\partial \tilde{I}_{1} }}\left( {J^{{{{ - 2} \mathord{\left/ {\vphantom {{ - 2} 3}} \right. \kern-0pt} 3}}} {\mathbf{1}} - \frac{1}{3}\tilde{I}_{1} {\mathbf{C}}^{ - 1} } \right) \\ & \;{ = }\frac{K}{{2 + r\left( {r + 1} \right)}}\left[ {2J\left( {J - 1} \right) - rJ^{ - 1} + rJ} \right]{\mathbf{C}}^{ - 1} + G\exp \left[ {\alpha_{1} \left( {\tilde{I}_{1} - 3} \right)} \right]\left( {J^{{{{ - 2} \mathord{\left/ {\vphantom {{ - 2} 3}} \right. \kern-0pt} 3}}} {\mathbf{1}} - \frac{1}{3}\tilde{I}_{1} {\mathbf{C}}^{ - 1} } \right) \\ \end{aligned}$$

12 $${\mathbf{S}}_{b}^{0} \left( {I_{4} } \right) = 2\frac{{\partial W_{b} }}{{\partial {\mathbf{C}}}} = 2\frac{{\partial W_{b} }}{{\partial I_{4} }}\frac{{\partial I_{4} }}{{\partial {\mathbf{C}}}} = 2\frac{{\partial W_{b} }}{{\partial I_{4} }}{\mathbf{b}}_{0} \otimes {\mathbf{b}}_{0} = 2\frac{{C_{4} }}{{\alpha_{4} }}\left\{ {\exp \left[ {\alpha_{4} \left( {I_{4} - 1} \right)} \right] - 1} \right\}{\mathbf{b}}_{0} \otimes {\mathbf{b}}_{0}$$

13 $${\mathbf{S}}_{c}^{0} \left( {I_{6} } \right) = 2\frac{{\partial W_{c} }}{{\partial {\mathbf{C}}}} = 2\frac{{\partial W_{c} }}{{\partial I_{6} }}\frac{{\partial I_{6} }}{{\partial {\mathbf{C}}}} = 2\frac{{\partial W_{c} }}{{\partial I_{6} }}{\mathbf{c}}_{0} \otimes {\mathbf{c}}_{0} = 2\frac{{C_{6} }}{{\alpha_{6} }}\left\{ {\exp \left[ {\alpha_{6} \left( {I_{6} - 1} \right)} \right] - 1} \right\}{\mathbf{c}}_{0} \otimes {\mathbf{c}}_{0}$$

14 $${\dot{\mathbf{q}}}_{iso}^{i} + \frac{1}{{\tau_{iso}^{i} }}{\mathbf{q}}_{iso}^{i} = \frac{{\gamma_{iso}^{i} }}{{\tau_{iso}^{i} }}{\mathbf{S}}_{iso}^{0}$$

15 $${\dot{\mathbf{q}}}_{b}^{i} + \frac{1}{{\tau_{b}^{i} }}{\mathbf{q}}_{b}^{i} = \frac{{\gamma_{b}^{i} }}{{\tau_{b}^{i} }}{\mathbf{S}}_{b}^{0}$$

16 $${\dot{\mathbf{q}}}_{c}^{i} + \frac{1}{{\tau_{c}^{i} }}{\mathbf{q}}_{c}^{i} = \frac{{\gamma_{c}^{i} }}{{\tau_{c}^{i} }}{\mathbf{S}}_{c}^{0}$$

where ⊗ denotes the dyadic operation between vectors, while $\gamma_{iso}^{i}$ and $\tau_{iso}^{i}$, $\gamma_{b}^{i}$ and $\tau_{b}^{i}$, $\gamma_{c}^{i}$ and $\tau_{c}^{i}$ are the viscous parameters associated to isotropic contribution and anisotropic terms.

##### Inverse Analysis of Experimental Tests and Identification of Constitutive Parameters

The analytical inverse analysis of the experimental tests first requires the development of formulations, based on the constitutive model, to interpret the stress states observed in the tests. It was convenient to refer to the first Piola-Kirchhoff stress tensor ${\mathbf{P}} = {\mathbf{FS}}$, being ${\mathbf{F}}$ the deformation gradient [35]. In fact, ${\mathbf{P}}$ directly interprets the nominal stress components that were measured during the experimental tests.

Considering first the equilibrium response obtained by processing the results of the stress-relaxation tests, the constitutive formulation can be elaborated in terms of nominal stress [36]:

17 $${\mathbf{P}}^{\infty } = \left( {1 - \sum\limits_{i = 1}^{{n_{iso} }} {\gamma_{iso}^{i} } } \right){\mathbf{FS}}_{iso}^{0} + \left( {1 - \sum\limits_{i = 1}^{{n_{b} }} {\gamma_{b}^{i} } } \right){\mathbf{FS}}_{b}^{0} + \left( {1 - \sum\limits_{i = 1}^{{n_{c} }} {\gamma_{c}^{i} } } \right){\mathbf{FS}}_{c}^{0}$$

With respect to a uniaxial stress state along ${\mathbf{a}}_{0}$, ${\mathbf{b}}_{0}$, or ${\mathbf{c}}_{0}$ direction, the deformation gradient takes a diagonal form:

18 $${\mathbf{F}} = \left[ {\begin{array}{*{20}c} {\lambda_{a} } & 0 & 0 \\ 0 & {\lambda_{b} } & 0 \\ 0 & 0 & {\lambda_{c} } \\ \end{array} } \right]$$

where $\lambda_{a}$, $\lambda_{b}$ and $\lambda_{c}$ are the stretch along the ${\mathbf{a}}_{0}$, ${\mathbf{b}}_{0}$, ${\mathbf{c}}_{0}$ directions, respectively. During a uniaxial test, e.g., along the ${\mathbf{a}}_{0}$ direction, the stretch $\lambda_{a}$ is imposed. The calculation of the stress component $P_{a}^{\infty }$ also requires knowledge of the lateral stretch components $\lambda_{b}$ and $\lambda_{c}$. These can be found by solving the system of non-linear equations given by setting the lateral stress components $P_{b}^{\infty }$ and $P_{c}^{\infty }$ to zero [38]. An equivalent approach applies to tests along the PA and LM directions.

The analysis of the normalized stress relaxation curves was performed using an exponential decay law, which was obtained by imposing the constant strain condition within the general constitutive formulation (Eqs. 10–13) and solving the differential Eqs. 14-16 describing the evolution of the viscous variables [31]. Assuming that the mechanical response along the ${\mathbf{a}}_{0}$, ${\mathbf{b}}_{0}$, ${\mathbf{c}}_{0}$ directions was predominantly determined by the stress contributions ${\mathbf{S}}_{iso}$, ${\mathbf{S}}_{b}$ and ${\mathbf{S}}_{c}$ respectively, the normalized relaxation curves can be interpreted by the following expressions:

19 $$p_{a} \left( t \right) = 1 - \sum\limits_{i = 1}^{{n_{iso} }} {\gamma_{iso}^{i} \exp \left( { - \frac{t}{{\tau_{iso}^{i} }}} \right)}$$

20 $$p_{b} \left( t \right) = 1 - \sum\limits_{i = 1}^{{n_{b} }} {\gamma_{b}^{i} \exp \left( { - \frac{t}{{\tau_{b}^{i} }}} \right)}$$

21 $$p_{c} \left( t \right) = 1 - \sum\limits_{i = 1}^{{n_{c} }} {\gamma_{c}^{i} \exp \left( { - \frac{t}{{\tau_{c}^{i} }}} \right)}$$

where *t* is time and $p_{a}$, $p_{b}$ and $p_{c}$ are the normalized stresses along the ${\mathbf{a}}_{0}$, ${\mathbf{b}}_{0}$, ${\mathbf{c}}_{0}$ directions.

Finally, to interpret uniaxial loading tests performed at a constant strain rate, it was necessary to consider the stress expressions (10-13), while the evolution of the viscous variable during loading was evaluated by numerically integrating the differential Eqs. 14-16.

In order to identify the constitutive parameters, the equilibrium response and stress relaxation processes were analyzed. Specifically, two cost functions were introduced to express the discrepancy between experimental and model results in the two cases. The first depended on the hyperelastic parameters, while the second depended on the viscous parameters:

22 $$\Omega^{\infty } \left( {{{\boldsymbol{\upomega}}}^{hyp} } \right) = \sum\limits_{j = 1}^{{m^{\infty } }} {\left\| {{\mathbf{E}}_{j}^{exp} - {\mathbf{E}}_{j}^{mod} \left( {{{\boldsymbol{\upomega}}}^{hyp} } \right)} \right\|} + \Gamma^{\infty } \left( {{{\boldsymbol{\upomega}}}^{hyp} } \right)$$

23 $$\Omega^{sr} \left( {{{\boldsymbol{\upomega}}}^{visco} } \right) = \sum\limits_{j = 1}^{{m^{sr} }} {\left\| {{\mathbf{R}}_{j}^{exp} - {\mathbf{R}}_{j}^{mod} \left( {{{\boldsymbol{\upomega}}}^{visco} } \right)} \right\|} + \Gamma^{sr} \left( {{{\boldsymbol{\upomega}}}^{visco} } \right)$$

where ${{\boldsymbol{\upomega}}}^{hyp} = \left[ {K \, r \, G \, \alpha_{1} \, C_{4} \, \alpha_{4} \, C_{6} \, \alpha_{6} } \right]$ and ${{\boldsymbol{\upomega}}}^{visco} = \left[ {\gamma_{iso}^{i} \, \tau_{iso}^{i} \, \gamma_{b}^{i} \, \tau_{b}^{i} \, \gamma_{c}^{i} \, \tau_{c}^{i} } \right]$ are the set of hyperelastic and viscous parameters, respectively, index *j* indicates the experimental curve among the $m^{\infty }$ equilibrium and $m^{sr}$ relaxation conditions studied, ${\mathbf{E}}_{j}^{exp}$ and ${\mathbf{R}}_{j}^{exp}$ are the vectors of experimental results from equilibrium and relaxation curves, respectively, ${\mathbf{E}}_{j}^{mod}$ and ${\mathbf{R}}_{j}^{mod}$ are the vectors of results from equilibrium and relaxation models, respectively, while ∥∥ is the vector modulus operation. The functions $\Gamma^{\infty }$ and $\Gamma^{sr}$ are penalty terms that allowed to introduce constraints on the values the constitutive parameters can take [39], such as positivity of the hyperelastic initial stiffness parameters.

To identify the optimal sets of hyperelastic and viscous parameters, it is necessary to minimize the respective cost functions, which exhibit multimodal behavior. Therefore, a stochastic-deterministic optimization algorithm based on the coupling of Simulated Annealing [40] and Nelder-Mead [41] methods was implemented and applied [38].

Once the optimal constitutive parameters had been identified, reliability was assessed by analyzing constant strain rate loading tests and comparing model and experimental results.

### Statistical Analysis

Continuous variables are reported as mean ± standard deviation. Data distribution normality was assessed using the Shapiro–Wilk test. Depending on the distribution, either an unpaired t-test or the Mann-Whitney test was applied. Statistical analysis was conducted using Minitab® 21.4.3. A p-value < 0.05 was considered statistically significant.

## Results

### Experimental Tests

Mechanical tests were performed to study the non-linear and time-dependent response of the plantar skin, examining the regional and directional tissue variability. The strain was computed as the ratio between the displacement measured by the testing machine (∆L) and the initial length/height (L_0_) of each individual sample, measured prior to testing. The stress was computed as the ratio between the force (F) measured by the load cell and the initial cross-sectional area (A_0_) of each individual sample. The A_0_ for dog-bone shaped samples was calculated as thickness×width*,* with both dimensions measured for each sample prior to testing, while for the cylindrical ones as $\pi \times {R}^{2}$ (where R is the radius, equal to half of the 5 mm of punch diameter). The mean stress–strain curves reported in the results were obtained by averaging the individual stress–strain curves calculated for each sample.

**Fig. 3:**
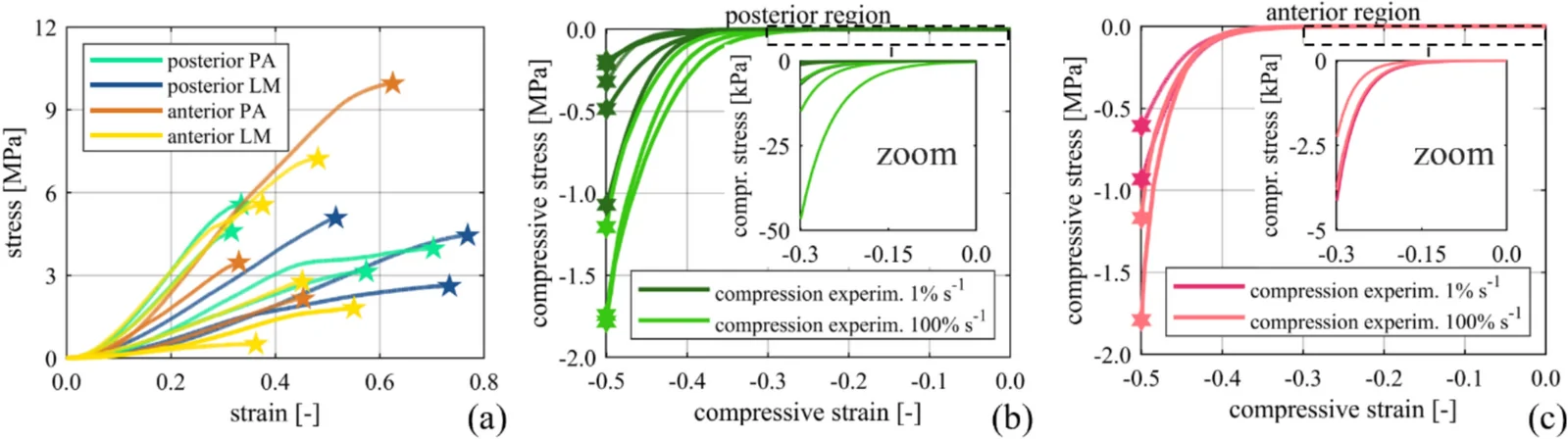
Example of curves obtained for one of the four tested subjects at **a** failure (the star represents the ultimate tensile stress and the corresponding failure strain); **b**, **c** compression at the two strain rates (1% s^−1^ and 100% s^−1^) for the posterior and anterior region of the foot respectively (the hexagram symbol represents the peak stress), inside a zoom showing the curves within strain range − 30%-0

Regarding the failure results, for all tested tissues, the stress-strain curves were considered up to the point of maximum stress, which was assumed as the failure point of the tissue. This point corresponds to the peak of the stress–strain curve, after which a marked decrease in load-bearing capacity was observed. The stress and strain values at this point were extracted as the ultimate tensile strength (UTS) and the failure strain (FS) to characterize the mechanical tensile response of the different tissues (represented with a star in Fig. 3a). The elastic modulus E_t_ was extracted from the linear region of the stress-strain curves (Table 2). Among all, the highest E_t_ and UTS were exhibited by PA samples from the posterior region (22.05 MPa and 6.63 MPa, respectively). More in detail, PA direction showed higher stiffness than LM direction in both posterior and anterior regions (posterior: 22.05 vs 12.91 MPa; anterior: 17.39 vs 12.82 MPa), even if, no significant differences were obtained. The FS was similar for each group, while the UTS were higher for PA direction concerning LM direction (Table 2). No significant differences were obtained between the posterior and anterior regions for each mechanical parameter.

**Table 2:** Mechanical parameters from tensile tests at failure

		POSTERIOR		ANTERIOR		P vs A
Variable	direction	Mean	SD	Mean	SD	p-value
UTS (MPa)	PA	6.63	4.22	6.22	3.44	0.891
	LM	4.77	2.17	4.52	2.34	0.600
	p-value	0.209		0.091		
FS (-)	PA	0.48	0.18	0.50	0.13	0.659
	LM	0.50	0.19	0.54	0.15	0.600
	p-value	0.572		0.600		
E_t_ (MPa)	PA	22.05	16.89	17.39	9.08	0.843
	LM	12.91	6.27	12.82	7.91	0.699
	p-value	0.125		0.117		

UTS, ultimate tensile strength; FS, failure strain; E_t_, elastic modulus at tensile; PA, posterior-anterior; LM, lateral-medial; P, posterior; A, anterior; SD, standard deviation.

Regarding the unconfined compression tests at different strain rates, the peak stress (PS), as the minimum stress reached by the sample at − 50% strain (represented with an hexagram symbol in Fig. 3b-c), and the final compression elastic modulus (E_c_), as the slope of the curve in the compression strain range of − 45 ÷ − 50%, were obtained (Table 3) from the reported stress-strain curves (Fig. 3b-c). The significant difference between the two strain rates of the PS (− 0.58 vs − 1.38 MPa, p = 0.002) and the E_c_ (6.09 vs 14.86 MPa, p < 0.001) for the posterior region confirmed the time-dependent response of the plantar skin. An increment of the same mechanical parameters with the increase in the strain rate was observed also for the anterior region, even if no statistical differences were obtained. An additional significant difference was observed between the posterior and anterior regions for E_c_ and PS at the lowest strain rate (p = 0.017 for peak stress and p = 0.005 for E_c_). Although stress values at up to 30% strain appear close to zero, they are actually low rather than zero, with measured stress values twice the load cell resolution, becoming detectable at approximately 20% strain. This behavior indicates a highly compliant mechanical response of the tissue within this strain range, as can be appreciated from the zoom panel in Fig. 3b and 3c. The near-zero stress readings are thus not due to measurement errors but are inherent to the material's mechanical properties, consistent with previous research on similar tissues [25, 42].

**Table 3:** Mechanical parameters from compression tests at different strain rates

		POSTERIOR		ANTERIOR		P vs A
Variable	strain rate	Mean	SD	Mean	SD	p–value
PS (MPa)	${\dot{\varepsilon }}_{1}$	–0.58	0.47	–0.99	0.59	0.017
	${\dot{\varepsilon }}_{2}$	–1.38	0.53	–1.52	1.09	0.277
	p–value	0.002		0.277		
E_c_ (MPa)	${\dot{\varepsilon }}_{1}$	6.09	4.10	13.24	7.33	0.005
	${\dot{\varepsilon }}_{2}$	14.86	4.71	17.04	11.79	0.591
	p–value	< 0.001		0.415		

PS, peak stress; E_c_, elastic modulus at compression; SD, standard deviation; P, posterior; A, anterior.

Data from stress–relaxation tests were analyzed to evaluate the material response during and after the relaxation phases. From the stress–time data, relaxation curves corresponding to each imposed strain step were extracted. To enable comparison under identical strain conditions, the stress values were normalized by calculating the ratio between the stress at each time point and the initial stress at the onset of the relaxation phase. Moreover, original stress–time data were processed to analyze the tissue mechanical response at the end of relaxation phases when all the time-dependent phenomena completely occurred. The equilibrium relative stiffness, as the value at the end of the relaxation phase, was computed for each curve (γ_∞_). No significant differences were obtained between anterior vs posterior regions or PA vs LM directions, while significant differences were obtained between PA vs CC direction and LM vs CC directions in each region (p < 0.001, Table 4).

**Table 4:** Equilibrium relative stiffness from tensile and compression at SR

		POSTERIOR		ANTERIOR		P vs A
Variable	direction	Mean	SD	Mean	SD	p-value
γ_∞_ (-) tensile SR	PA	0.39	0.03	0.41	0.04	0.163
	LM	0.40	0.06	0.38	0.03	0.736
γ_∞_ (-) compression SR	CC	0.08	0.02	0.07	0.03	0.173
	p-value PA vs LM	0.736		0.112		
	p-value LM vs CC	** < 0.001**		** < 0.001**		
	p-value PA vs CC	** < 0.001**		** < 0.001**		

SR, stress-relaxation, where the stress is normalised to the initial stress value; PA, posterior-anterior; LM, lateral-medial; CC, cranial-caudal; P, posterior, A, anterior; SD, standard deviation.

By considering the stress at the equilibrium point for each applied strain step, equilibrium stress–strain curves were obtained for each specimen by interpolating these points using an exponential function.

**Fig. 4:**
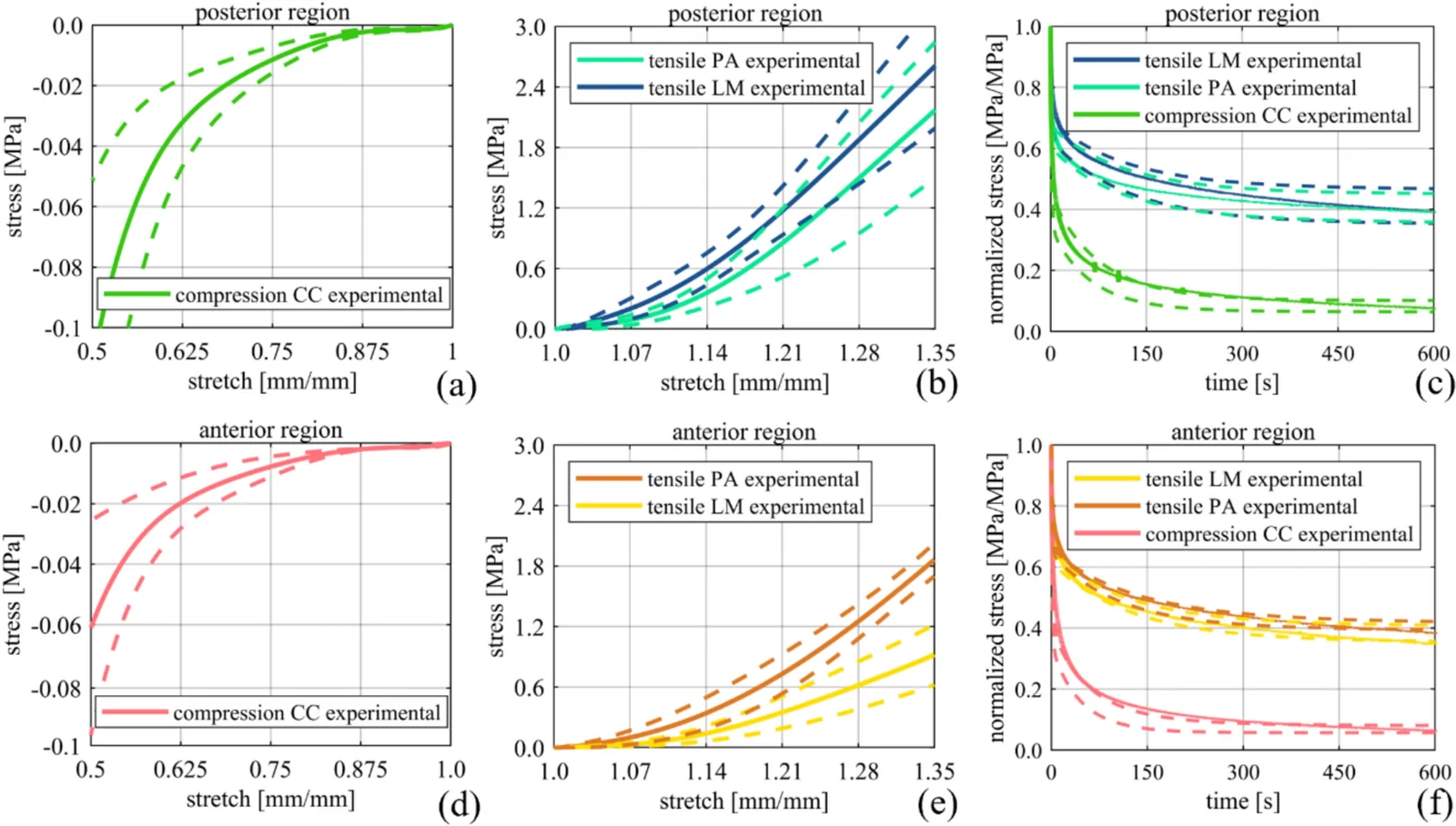
Results of stress relaxation experimental tests on skin specimens. Equilibrium stress-stretch curves (on the left) and normalized stress-time curves (on the right) for the posterior (**a**-**c**) and anterior (**d**-**f**) regions considering both compressive (carried out along the CC direction) and tensile (performed on PA and LM directions). Loading stress-stretch data are represented as mean ± SD, while for the normalized stress-time curves, an example of curves for each direction is reported

For the constitutive analysis, stretch has been derived from the strain ($\lambda =\varepsilon +1$), Fig. 4 presents the equilibrium stress–stretch curves and the normalized stress–time curves for the anterior (a–c) and posterior (d-f) regions.

### Identification and Validation of Constitutive Parameters

**Fig. 5:**
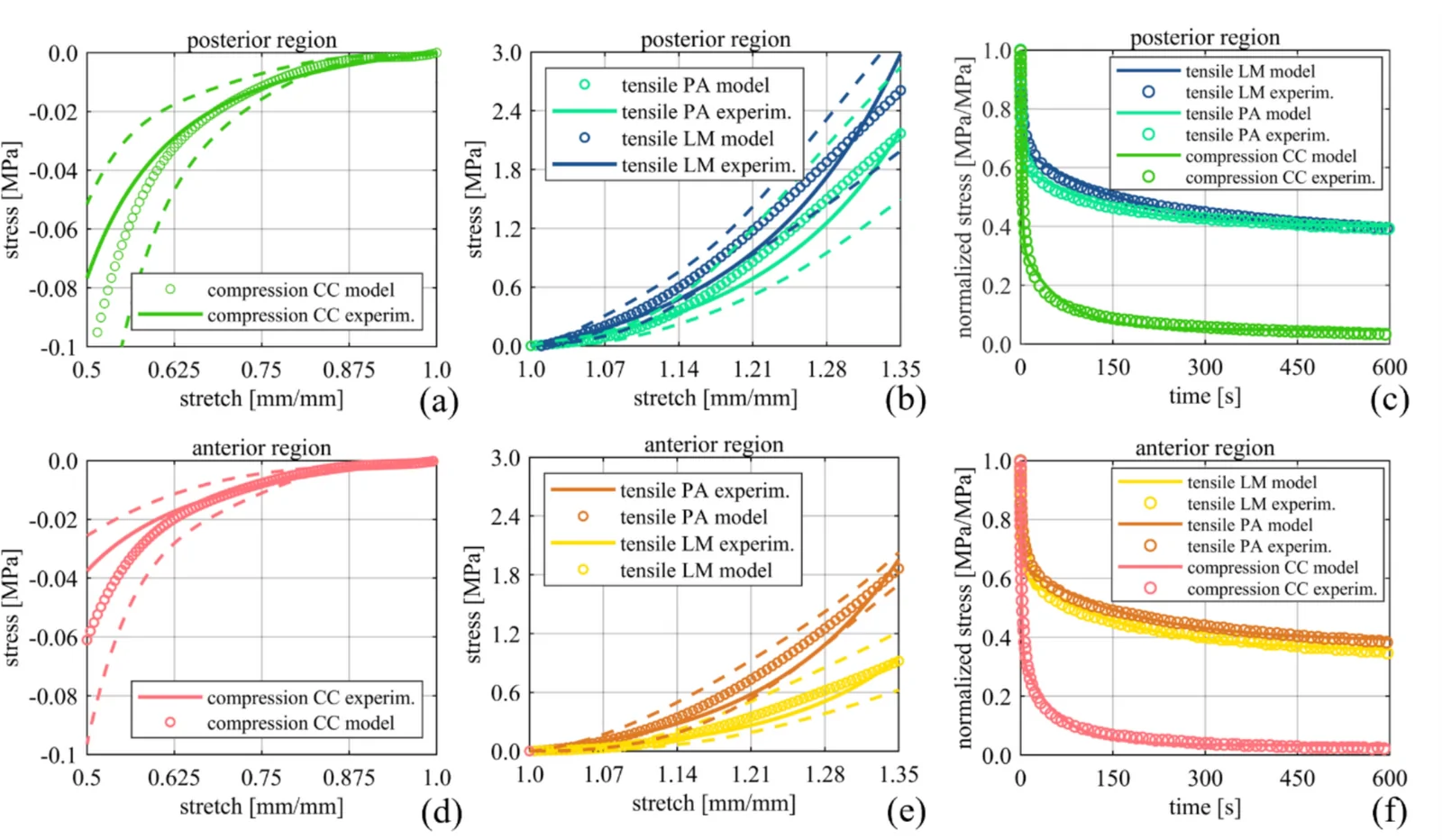
Comparison of experimental and constitutive model results. Identification of constitutive parameters by inverse analysis of equilibrium stress-stretch curves and normalized stress relaxation curves for the posterior (**a-c**) and anterior region of the foot (**d-f**). Stress-stretch curves are reported as mean ± SD, while the normalized stress-time ones are an example of experimental results with their corresponding model curve achieved through inverse analysis

**Fig. 6:**
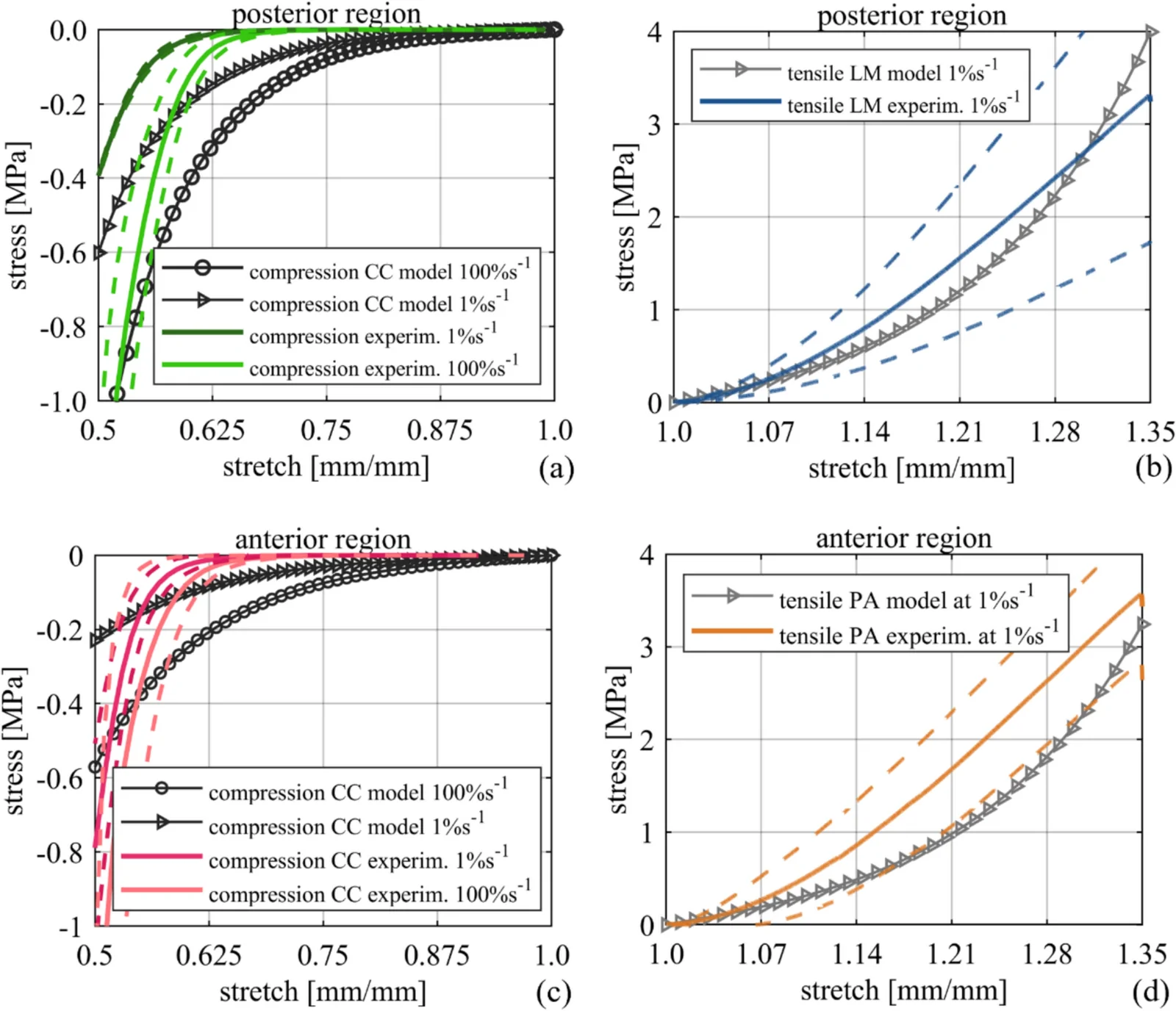
Comparison of experimental and constitutive model results. Reliability assessment of parameters through analysis of constant strain rate compression and tension tests: **a**, **b** posterior and **c**, **d** anterior region. Stress-stretch curves are reported as mean ± SD

To implement the plantar skin tissue in a computational model of the foot, an anisotropic visco-hyperelasticity model with two viscous branches was adopted. The constitutive model, with its hyperelastic (Table 5) and viscous (Table 6) parameters, was able to interpret the behavior of the tissue in the posterior and anterior regions, in terms of the anisotropic, non-linear and time-dependent response (Figs. 5 and 6). To evaluate the discrepancy between the experimental data and the model results, the normalised mean absolute percentage error procedure (WMAPE), in terms of loads, was adopted. Concerning the equilibrium stress-strain curves obtained through SR tensile and compression tests, the value of discrepancy computed was about 16% for the anterior region and 19% for the posterior ones.

**Table 5:** Hyperelastic constitutive parameters

Region	K_v_[kPa]	r [-]	G[kPa]	α_1_[-]	C_4_[kPa]	α_4_[-]	C_6_[kPa]	α_6_[-]
posterior	24.50	2.60	2.50	2.35	350.00	2.20	500.01	2.10
anterior	21.20	1.69	1.50	1.60	280.00	2.40	118.90	2.64

**Table 6:** Viscous constitutive parameters

Region	Viscous component	γ_1_[-]	γ_2_ [-]	τ_1_[s]	τ_2_[s]
posterior	q^i^_b_	0.38	0.23	1.78	115.94
	q^i^_c_	0.36	0.25	1.87	118.97
	q^i^_a_	0.61	0.31	1.98	70.93
anterior	q^i^_b_	0.35	0.24	1.84	129.93
	q^i^_c_	0.37	0.24	2.10	127.19
	q^i^_a_	0.63	0.30	1.66	56.61

The validation of the constitutive model and parameters was obtained considering the tensile at 1%s^−1^ and compression tests at 1%s^−1^ and 100%s^−1^. The analytical stress-strain results fall inside the mean curve ± standard deviation for both the posterior and anterior regions, showing the capability of the model to interpret the viscous phenomena and the anisotropic behavior. In this case, the percentage error values range between 37% (for the anterior region) and 43% (for the posterior region).

## Discussion

There are several methods adopted to test skin using in vivo and ex vivo procedures. Most of the literature investigating the mechanical behavior of skin concentrates on the skin of main body areas (e.g., the back, forearm, thigh [43–46], and the few studies that also examine plantar skin do so mainly through in vivo tests concentrating on diseased tissue [47, 48]. While in vivo measurements, which can be classified as static (single modulating stimulus) or dynamic (cyclic stimulus), are crucial since they consider both skin function and its interaction with the surrounding tissues, ex vivo tests provide information on tissue properties using repeatable and standardized methods [49]. Furthermore, they provide information on the properties of the skin itself, without being influenced by the response of the underlying tissues, such as adipose tissue, plantar fascia, muscles, or even bones. Concerning ex vivo tests, to the best of the authors' knowledge, this is one of the first studies in which plantar skin has been studied using different loading conditions and the experimental results have been used to develop an anisotropic visco-hyperelastic constitutive model that describes the mechanical response, including the identification and validation of its parameters.

Tensile tests up to failure demonstrate the plantar skin's non-linear anisotropic behavior. Results from both tensile and compression tests at constant strain rate highlight to assert the strain rate dependence behavior of the plantar skin tissue. Stress-relaxation results, from both tensile and compression loading, confirm the tissue’s time-dependent behavior, showing the capability of the tissue to reach the equilibrium in 600 s.

Upon examining the results in greater detail, various considerations can be made, taking into account the two-foot regions (posterior and anterior) and the sample directions (PA and LM). Tensile tests, conducted up to failure, show no statistically significant difference between the regions or the two sample directions. Nevertheless, it is possible to observe some trends among the mechanical parameters. In particular, the PA direction exhibits an E_t_ and UTS greater than those in the LM direction (for both regions), although it breaks at FS lower than those of the LM direction. Slightly different behavior was observed in the compression tests at different strain rates (loading-unloading protocol). In this case, the PS and E_c_ parameters are greater in the anterior region compared to the posterior region, and they statistically differ at the lowest strain rate (p = 0.017 and p = 0.005 for the peak stress and E_c_, respectively). Another characteristic is that the values of the above-mentioned parameters increase with increasing strain rates. This property is more evident for the posterior foot region, where the PS increases in modulus from 0.58 MPa at ${\dot{\varepsilon }}_{1}$ to 1.38 MPa at ${\dot{\varepsilon }}_{2}$(p = 0.002), and the E_c_ rises from 6.09 MPa at ${\dot{\varepsilon }}_{1}$ to 14.86 MPa at ${\dot{\varepsilon }}_{2}$(p < 0.001). Results also allowed for the assessment that the CC direction exhibits markedly different viscoelastic behavior compared to the PA and LM ones (p < 0.001). The stress relaxation experimental results (both tensile and compressive) enabled to determine, through inverse analysis, the constitutive parameters of the anisotropic visco-hyperelastic model for the posterior and anterior regions (Tables 5 and 6), while the loading-unloading unconfined compression and tensile tests, including data up to failure, were used to validate the model parameters (Fig. 6), showing a percentage error of about 37–43%.

Taken together, these findings indicate that the tensile mechanical behavior is consistent across foot regions and orientations, although Young’s modulus and UTS are higher in the PA direction and in the posterior regions. In contrast, compression properties vary significantly between posterior and anterior regions. Although the different cleavage line distribution across the foot have been assessed during the past decades, leading to a non-clear and univocal identification of collagen orientation, Langer lines appear to better represent the collagen fibers distribution according to the present experimental results achieved, since they are circumferentially organized in the heel pad and 1^st^ metatarsal regions, while they are transversally arranged in the other foot locations [11]. Furthermore, the observed variations in mechanical properties likely reflect functional adaptations of plantar skin to local mechanical loading to which the foot is subjected during standing and gait, suggesting that plantar skin is regionally tuned to withstand habitual loads [3].

The comparison between the results achieved and those in the literature is limited, since very few works have investigated the mechanical behavior of human skin, with almost all focusing on non-plantar skin tissue. Comparing our findings with existing results, it is possible to observe that the back skin tested in [43, 44] showed a higher elastic modulus, UTS, and FS than those reported in this work, but of the same order of magnitude. On the other hand, our values fall within the range proposed by [50], which averaged the parameter values obtained from samples of various anatomical skin parts, and show higher values in terms of elastic modulus than those proposed by Wahlsten et al. [51], which considered breast and abdomen skin. Considering the similar anatomical site (foot region), Crossland et al. [9] tested the calcaneal region up to failure, while Bradi et al. [10] carried out compression tests on samples harvested from six different plantar regions (hallux, first, third, and fifth metatarsal heads, lateral midfoot, and calcaneus). Both studies report lower mechanical parameters than those observed in our study. Indeed, Crossland et al. [9] report a final elastic modulus of 0.83 ± 0.49 MPa and a UTS of 0.17 ± 0.07 MPa, while Brady et al. [10] determined a final elastic modulus in compression of 1.79 ± 0.13 MPa. The discrepancies mentioned above could likely be attributed to the different skin regions considered (foot vs. back or abdomen) or the slightly different strain rate used in the tensile failure protocol. Above all, the most important one, which can alter the mechanical properties more than the anatomical skin region, is the samples' origin. Considering this last point, most existing research uses cadaver tissue, whereas ours derives from fresh, living patients. Cadaver samples often undergo post-mortem changes and repeated freeze–thaw cycles, which are known to modify soft-tissue mechanics. These differences in tissue preservation and testing procedures likely contribute to the variations observed in mechanical properties measurements.

The mechanical properties discussed in this study can be compared to those of commercial artificial skin models (ASMs), which are used to repair injuries, including diabetic foot ulcers (DFUs). Kho et al., (2023) [18] analysed four types of ASMs through hardness, needle insertion, and tensile and compression tests. Considering the mechanical parameters obtained through the tensile test, the ASM exhibits UTS and failure strain values ranging from 1.35 to 8 MPa and from 2 to 20%, respectively. While the UTS values are comparable to those obtained for the plantar skin, the failure strain exhibited by the artificial skin is lower, highlighting that artificial skin substitute research is on the right path, even if some improvements are still needed.

Over the past few years, numerous studies have sought to define constitutive models capable of describing the mechanical behavior of human skin. One of the simplest approaches focused solely on capturing the non-linear stress–strain relationship of lower back skin using an incompressible isotropic Ogden model [52]. Other studies have also accounted for the anisotropic contribution of collagen fibres, typically employing incompressible, anisotropic hyperelastic models to represent skin in regions such as the back, abdomen, breast, and face. These models, which have demonstrated good predictive capability, generally couple an isotropic neo-Hookean model for the non-collagenous ground matrix with a Gasser–Ogden–Holzapfel (GOH) model for two fibre families oriented according to the mean direction of Langer lines [12, 44, 52–56]. Some variation of these formulations has also been proposed; for instance, Alliliche et al. (2023) [57] introduced a Yeoh model for the isotropic matrix component, rather than the Neo-Hookean ones. Despite their accuracy, these models present several limitations. First, most models assume skin to be incompressible. However, Wahlsten et al. (2019) [58], through uniaxial tests on abdominal and breast skin, observed up to 30% volume loss at a stretch ratio of 1.2, attributed to interstitial fluid efflux. This suggests that compressibility should be considered, as done by Pond et al. (2018) [59], who adopted a compressible isotropic, Neo-Hookean model to describe the matrix behavior. Second, the vast majority of studies have focused on the back, abdominal, breast, or facial skin, while very few have investigated the mechanical behavior of plantar skin. An exception is the model developed by the authors’ research group [24], which employed a fibre-reinforced hyperelastic formulation. However, its parameters were derived from inverse analyses of pig skin experiments (considered to show similar mechanical properties to the human) [60, 61], limiting its applicability due to the lack of data on human plantar tissue. Third, existing models largely neglect the viscous component, despite the well-documented time-dependent behavior of skin [8, 46, 62, 63]. Therefore, the anisotropic visco-hyperelastic constitutive model proposed in this study provides a comprehensive formulation to interpret the mechanical response of plantar skin, as characterised through a series of specific experimental procedures. The model captures the main visco-hyperelastic and anisotropic features observed experimentally; however, the WMAPE values (16–43%) indicate limited accuracy in pointwise stress prediction, suggesting possible improvements to the constitutive formulation. This is one of the aspects of the current work that should be considered and addressed in future developments.

Another limitation concerns the lack of histological images, which would help to investigate the arrangement of collagen fibers, thus providing additional information to interpret the results achieved along the CC, PA, and LM directions. It should also be noted that the mechanical tests in this study do not necessarily align with the principal directions of anisotropy of the plantar skin; however, due to the lack of precise information on the collagen fibre arrangement, the two loading directions have been assumed as the principal directions in the constitutive model. This represents an additional limitation of the current study, which should be considered in future work. Another methodological issue is that stress and strain were reported in their engineering (nominal) formulation, as full-field strain measurements, such as digital image correlation (DIC), were not used. Although the dog-bone specimen shape encourages deformation to concentrate in the gauge area, nominal stress and strain differ from the actual true stress and local strain fields. Additionally, the absence of lateral strain data hampers direct experimental evaluation of transverse deformation, which could be especially important for anisotropic tissues. Another methodological aspect concerns sample hydration during testing. The adopted procedure, consisting of periodic pipetting of saline solution at intervals of no more than 300 s, was intended to minimise surface drying; however, some degree of dehydration cannot be completely excluded. Although complete sample immersion in a temperature-controlled hydration bath has been adopted in tensile testing of human breast skin to better preserve physiological conditions [64], this approach was not suitable in the present study. Indeed, the overall testing period for the stress relaxation procedure (both tensile and compression) exceeded 50 minutes, and prolonged water exposure has been reported to alter the mechanical properties of plantar skin after approximately 45 minutes [2]. Therefore, continuous immersion might have introduced additional alterations in tissue behaviour. Nevertheless, all specimens were tested under the same hydration protocol, ensuring internal consistency in the comparative analyses, even though further investigation could be performed in the future. An additional aspect to consider is the limited number of human subjects involved in the study, resulting in a small sample size (N = 4) from which samples were harvested. It will be interesting to expand the patient cohort so that other experimental tests can be performed (*e.g.*, shear tests) and the investigation of a possible relationship between subject characteristics (such as age, sex, and BMI) can be conducted. Finally, the involvement of diabetic patients could be insightful. The development of experimental tests on diseased skin tissue and then the mechanical investigation of its properties will be crucial for understanding the differences between healthy and diseased conditions, maybe providing new information on the mechanism of diabetic plantar ulcers' onset and evolution.

The experimental findings indicated varying mechanical characteristics in different plantar skin areas and directions. The results obtained enhance our understanding of foot skin behavior, providing its complete mechanical characterization, such as elastic modulus or UTS, which can be crucial for various applications. These include diagnosing and monitoring skin diseases, designing and developing skin substitutes, and quantifying the load tolerance of skin, which is essential for preventing skin injuries. A visco-hyperelastic constitutive model and the associated constitutive parameters have been identified that be beneficial in developing in silico tools for the foot. These tools could be valuable in evaluating and simulating the effects of various conditions, *e.g.*, diabetes, as well as in exploring the interactions between the foot and footwear.

## Data Availability

Available upon reasonable request to the corresponding author
